# Drinking Ether

**Published:** 1891-09

**Authors:** 


### Drinking Ether.

There has lately been a great outcry concerning the consumption of ether by the Irish who have chosen it as an intoxicant, and in the House of Commons, in answer to a question by Dr. Tanner, Mr. Balfour said: "As stated by my right honorable friend the Chancellor of the Exchequer, in reply to a question addressed to him on December 1st, ether has been scheduled and it can now only be sold by qualified chemists or druggists as a poison." The statement laid before the executive government by Mr. Thomas Ledlie afford some idea as to the alarming proportions the habit had assumed. Mr. Ledlie calculates that seventeen thousand gallons of impure ether of the vilest form is annually consumed by the people in the districts situated in the counties of Derry and Tyrone, as well as parts of Armagh, Monaghan and Fermanagh, no fewer than 100,000 people spread over an infected area of 190,000 acres indulge in this baneful habit. He suggests six ways of getting rid of what he considers a baneful practice, the chief of which may be summarized as follows: The introduction of naphtha into the preparation of all ether, save that used purely for medicinal purposes, which would give it nauseating odor and taste, the reimposition of a prohibitive tax, and the making of the practice illegal and placing it upon the criminal code, as well as making the sale of ether illegal by all all persons save chemists, and by this class of persons only for *bona fide* medicinal or commercial purposes.—*Letter to Am. Jour. Med. Ass'n.*

